# Gambling among employees in Swedish workplaces: A cross-sectional study

**DOI:** 10.1539/eohp.2022-0020-OA

**Published:** 2023-04-22

**Authors:** Jonas Rafi, Petra Lindfors, Per Carlbring

**Affiliations:** 1 Department of Psychology, Stockholm University, Stockholm, Sweden

**Keywords:** cluster randomized trial, gambling, prevention, problem gambling, workplace

## Abstract

**Objectives:**

Responsible workplaces strive to minimize the harmful effects of alcohol and drug abuse. However, gambling is still a neglected area in workplace research. This study describes workplace gambling and investigates variables associated with at-risk and problem gambling (ARPG) and knowing about colleagues who gambles during work, using cross-sectional data from a cluster-randomized controlled trial on gambling prevention in the workplace (N=3,629).

**Methods:**

Measures included ARPG and knowledge about colleagues who gamble during work.

**Results:**

Of the respondents, 168 (4.7%) knew of someone who gambles at work. Knowing about a colleague who gambles during work was more common among employees who were men (odds ratio [OR] 2.98; 95% confidence interval [CI], 2.07–4.29), aged 16–34 years (OR 1.97; 95% CI, 1.19–3.28), knew about a gambling policy (OR 1.57; 95% CI, 1.03–2.39), and who themselves were classified as ARPGs (OR 2.95; 95% CI, 1.60–5.35). Similarly, being classified as an ARPG was significantly associated with being a man (OR 2.14; 95% CI, 1.43–3.20), aged 16–34 (OR 2.35; 95% CI, 1.21–4.54) or 35–44 (OR 2.36; 95% CI, 1.30–4.27) years, being a subordinate (OR 2.53; 95% CI, 1.02–6.30), and knowing about a colleague who gambles during work (OR 4.02; 95% CI, 2.38–6.79).

**Conclusions:**

Gambling during work is a prevalent phenomenon. Organizations should consider implementing gambling policies that facilitate helping workers who are problem gamblers. To determine policy contents and measures to implement, the type of gambling and its effect on employees should be explored.

## Introduction

In Sweden, participation in gambling is generally high. In 2016, 29% of individuals who are in the age range at which people usually work in Sweden (16–67 years) gambled regularly, and 6% were classified as at-risk and problem gamblers (ARPGs)^1)^. In addition to the individual harm that can come from gambling, organizations can also be negatively affected by the behavior of ARPGs. These harms range from employees arriving late to work to monetary embezzlement^2,3)^. With the increasing use of smartphones to gamble, productivity issues are also of growing concern^4)^. Previous research has highlighted the importance of using workplace policies and appropriate responses to prevent any harm from gambling in the workplace^2,5)^. While the presence of alcohol and drug policies has been associated with lower use of alcohol and drugs^6)^, no studies have investigated the effect of gambling policies on the prevalence of workplace gambling. Despite this, it is not unreasonable to assume that the same holds for gambling. However, having a policy is only one side of the coin. To respond appropriately to workplace gambling, regardless of whether it is harmful or not, it is necessary to be aware of the amount of gambling that occurs. Gambling has been described as an “invisible addiction”^7)^ and is more challenging to identify than alcohol or drug addictions. Thus, the extent to which employees are aware of colleagues who gamble during work is valuable information to the organization. For example, most occupational fraud is discovered through a tip, which points up the need for employees to be willing to act in the interest of an organization^8)^.

In the current study, we used previously collected data to explore aspects of gambling relevant to prevention in a sample of employees in Sweden. More specifically, we investigated 1) managers’ and subordinates’ knowledge of the existence of workplace policies on gambling, gaming, alcohol, medications and drugs; 2) the prevalence and associations of knowing about colleagues who gamble during work; and 3) the prevalence and associations with ARPGs among different groups (ie, managers and subordinates).

## Methods

### Study design

This was a secondary exploratory analysis of cross-sectional self-report data collected in a cluster-randomized controlled trial^9)^. Ten organizations were randomized to receive either a workplace prevention program for problem gambling or serve as a wait-list control group. The organizations were recruited at a conference about harmful use, where the developers of the prevention program presented the results of their previous intervention and recruited organizations to participate in the randomized controlled trial. The prevention program consisted of policy implementation and skill-development training for managers. Subordinates were surveyed at baseline and 12 months post-baseline, and managers were surveyed at baseline and approximately 6- and 12-months post-baseline. Readers interested in the prevention program are referred to the study protocol^10)^. This study, however, focused exclusively on the baseline measurements. The Regional Ethics Committee in Stockholm approved this project (Ref. No. 2016/1208-31/5). All participants were informed about the study purpose and design and provided written informed consent.

### Participants

All individuals who were employed at any of ten organizations, at the time of data collection, were invited to participate (n=8,672). Of these, 4,270 failed to respond to the questionnaire, and 637 omitted data on one or more of the data variables^*1^. The remaining 3,629 (42%) respondents with complete data were included. All workplaces employed both blue- and white-collar workers, but this information was not available at the individual level. Table 1 describes the workplaces, and Table 2 describes respondent demographics.

**Table 1.** Description of organization sectors, work settings, and response rates

Organization number	Sector	Setting^a^	N employed at baseline (n=8,524)	N responded (n=3,629)
1	Public	Government Authority	808	630 (78%)
2	Public	Higher Education Institution	2,048	443 (21.6%)
3	Public	Higher Education Institution	852	320 (37.6%)
4	Private	Dairy Farming	413	246 (59.6%)
5	Public	Staffing Agency	598	269 (45%)
6	Private	Energy	205	145 (70.7%)
7	Public	Higher Education Institution	514	112 (21.8%)
8	Public	Healthcare	2,569	1,214 (47.2%)
9	Public	Higher Education Institution	430	181 (42.1%)
10	Private	Electronics Manufacturer	87	69 (79.3%)

a All workplaces included both blue- and white-collar workers.

**Table 2.** Description of demographic characteristics and data variables related to gambling and other harmful use in the workplace

	All employees (%)	Managers (%)	Subordinates (%)
**Gender**			
Man	1,415 (39.1)	177 (47.7)	1,238 (38.1)
Woman	2,179 (60.3)	194 (52.3)	1,985 (61.2)
Other	23 ( 0.6)	0 ( 0.0)	22 ( 0.7)
**Age group**			
16–34	504 (13.9)	10 ( 2.7)	494 (15.2)
35–44	998 (27.5)	100 (27.0)	898 (27.6)
45–54	1,157 (31.9)	146 (39.4)	1,011 (31.0)
55+	970 (26.7)	115 (31.0)	855 (26.2)
**Awareness of workplace policy for:**			
Gambling	408 (11.4)	40 (10.8)	368 (11.4)
Gaming	613 (17.0)	62 (16.7)	551 (17.1)
Alcohol	2,402 (66.5)	350 (94.3)	2,052 (63.3)
Drugs	1,784 (49.6)	273 (73.6)	1,511 (46.8)
Medications	948 (26.3)	160 (43.1)	788 (24.4)
**Know of a colleague who**			
Gambles during work	168 ( 4.7)	16 ( 4.3)	152 ( 4.7)
Games during work	547 (15.2)	52 (14.0)	495 (15.3)
**Worried about a colleague regarding**			
Gambling	62 ( 1.7)	13 ( 3.5)	49 ( 1.5)
Gaming	88 ( 2.4)	16 ( 4.3)	72 ( 2.2)
Alcohol	369 (10.2)	102 (27.5)	267 ( 8.2)
Drugs	47 ( 1.3)	13 ( 3.5)	34 ( 1.1)
Medications	109 ( 3.0)	37 (10.0)	72 ( 2.2)
**Knows who to contact about**			
Gambling	1,996 (55.4)	258 (69.5)	1,738 (53.8)
Gaming	1,986 (55.1)	257 (69.3)	1,729 (53.5)
Alcohol	2,626 (72.8)	364 (98.1)	2,262 (69.9)
Drugs	2,487 (69.0)	346 (93.3)	2,141 (66.3)
Medications	2,406 (66.8)	343 (92.5)	2,063 (63.8)
**PGSI**			
ARPG (PGSI >0)	128 ( 3.5)	5 ( 1.4)	123 ( 3.8)
Low risk (PGSI 1–2)	68 ( 1.9)	—	—
Medium risk (PGSI 3–7)	39 ( 1.1)	—	—
High risk (PGSI 8+)	21 ( 0.6)	—	—

Note. ^a^ PGSI risk group prevalence rates split by role is not presented to not present groups with n<5. ARPG: At-risk and problem gambler. PGSI: Problem Gambling Severity Index.

*1Of these, 24 chose the option “other” when asked to disclose their gender and were thus categorized as having missing data. This strategy was chosen since the group was too small to be analyzed separately and it is ethically questionable to assign a gender based on individuals’ names.

### Data collection

Outcome measure data were collected using a questionnaire administered to participants through Iterapi, a secure and encrypted online platform^11)^. E-mail reminders to participate were sent up to four times. Participants with no organizational email address received either a paper questionnaire or shared a workplace computer to access the questionnaire online. These participants did not receive any reminders.

### Data variables

The questionnaire was developed for the cluster-randomized controlled study and included both multiple independent questions aimed at covering specific aspects (such as policy knowledge) and the problem gambling severity index (PGSI)^12)^. The PGSI is a commonly used self-report measure, with sound psychometric properties, for measuring problem gambling in prevalence studies^13)^. The PGSI consists of 9 items, scored 0–3, measuring risk of problem gambling (eg, “Have you bet more than you could afford to lose?”). In this study, the PGSI total score (0–27) and four of the questions formulated for the cluster-randomized trial were included. Each of the four questions consisted of separate sub-questions for each area of harmful use. The questions were: 1) whether the participant knew about a colleague who gambled or gamed during work; 2) whether they were aware of their workplace having a policy for each of the areas gambling, gaming, alcohol, drugs, or medications; 3) whether they were worried about a colleague’s participation/use for each of the areas gambling, gaming, alcohol, drugs, or medications; and 4) whether they knew whom to contact over concerns about a colleague’s participation/use for each of the areas gambling, gaming, alcohol, drugs, or medications. The complete questionnaire is published elsewhere^10)^. The questionnaire items (except for PGSI) were coded as 0 for “No” or 1 for “Yes” in the analyses. Because of the low incidence of moderate-risk (PGSI scores of 3–7) and high risk (PGSI ≥8) gamblers, we adopted a previously used method^1)^ and included anyone with a non-zero PGSI score in the same group: at-risk and problem gambler (ARPG). The independent variables included were age group (16–34, 35–44, 45–54, and ≥55 years) gender (man, woman), and position at work (subordinate, manager). Age groups were used because actual age was not available. The type of work (eg, academia, administration) was not included in the models because each organization typically employed both blue- and white-collar staff. Also, the exact categories and job titles were frequently unavailable, precluding analyzing the difference between occupations.

### Data analysis

As the employees are nested within organizations and the outcome variables are binary, generalized hierarchical linear models were used, with organization included as a group-level covariate in all analyses to account for similarities within organizations. In the first model, the variable “knowing about a colleague who gambles during work” was set as dependent variable. Independent variables were age, gender, role, gambling policy, and ARPG. In the second model, the variable “ARPG” was set as dependent variable. Independent variables were age, gender, role, gambling, policy, and knowing about a colleague who gambles during work. Thus, the difference between the models was whether ARPG and knowing about a colleague who gambles during work were set as an independent or dependent variable. For the odds ratios (OR), the response alternative “no” was set as a reference level for being an ARPG, awareness or gambling policy, and awareness of collegial gambling. For age, gender, and position, the group with the lowest outcome incidence was set as the reference level. Data cleaning and analyses were conducted in R version 4.1.1 (R Foundation for Statistical Computing, Vienna, Austria)^14)^ using lme4^15)^ and tidyverse^16)^. Because the age groups of 16–24 years (n=48) and ≥65 years (n=61) were approximately a tenth of the size of the other age groups, they were merged with their closest age groups.

## Results

Demographic statistics and proportions for all variables are presented in Table 2. Of the respondents, 168 employees (4.7%) stated that they knew someone who gambles during working hours, and 29 of these (17.4%) also stated that they were worried about a colleague regarding negative consequences of gambling. As for PGSI, 128 respondents (3.5%) scored 1 or more and were thus classified as ARPGs (Table 2). One organization had no individuals reporting ARPG, the others ranged between 1.3 and 5.3 percent. The highest percentages of ARPG were found among organizations mainly consisting of blue-collar workers, such as dairy farming (5.3%) and staffing agency (4.7%).

The first regression analysis showed that being a man, categorized as an ARPG, and belonging to the 16–34-year age group were associated with knowing about a colleague who gambles during work (Table 3). There was no evidence of an association between position or the existence of a gambling policy and knowledge about a colleague who gambles during work.

**Table 3.** Associations between five independent variables and the outcome variable “knowing about a colleague who gambles during work”

	N (%)^a^	OR	CI	*p*
**Age group, years**				
16–34	41 ( 8.2%)	1.97	1.19–3.28	0.009
35–44	41 ( 4.1%)	1.23	0.75–2.01	0.408
45–54	52 ( 4.5%)	1.27	0.79–2.03	0.326
≥55	34 ( 3.5%)	ref		
**Gender**				
Man	114 ( 8.1%)	2.98	2.07–4.29	<0.001
Woman	48 ( 2.2%)	ref		
**Role**				
Subordinate	152 ( 4.7%)	1.13	0.65–1.96	0.673
Manager	16 ( 4.3%)	ref		
**Awareness of workplace gambling policy**				
Yes	33 ( 8.2%)	1.57	1.03–2.39	0.036
No	133 ( 4.2%)	ref		
**ARPG**				
Yes	25 (20.2%)	2.95	1.63–5.35	<0.001
No	143 ( 4.1%)	ref		

ARPG, at-risk and problem gamblers; CI, confidence interval; OR, odds ratio.

a Percentages indicate the fraction of individuals who know someone who gambles during work in each specific subgroup.

The second regression analysis showed that knowing someone who gambles during work, being a man, and belonging to the 16–34- or 35–44-year age groups were significantly associated with being an ARPG (Table 4). In contrast, there was no evidence of a difference in ARPG rates as a function of position or of stating the existence of a gambling policy. Among the 105 employees indicated as ARPGs, 62 (59%) were men. Age analyses were omitted due to the low incidence of problem gambling when splitting the sample into age groups.

**Table 4.** Associations between five independent variables and the outcome variable “at-risk and problem gambler (ARPG)”

	N (%)^a^	OR	95% CI	*p*
**Age group, years**				
16–34	28 (5.6%)	2.35	1.21–4.54	0.012
35–44	41 (4.1%)	2.36	1.30–4.27	0.005
45–54	33 (2.8%)	1.43	0.76–2.68	0.263
≥55	26 (2.7%)	ref		
**Gender**				
Man	69 (4.9%)	2.14	1.43–3.20	<0.001
Woman	48 (2.2%)	ref		
**Role**				
Subordinate	123 (3.8%)	2.53	1.02–6.30	0.046
Manager	5 (1.4%)	ref		
**Gambling policy exists**				
Yes	15 (3.7%)	0.85	0.47–1.55	0.604
No	107 (3.4%)	ref		
**Knows someone who gambles during work**				
Yes	25 (15%)	4.02	2.38–6.79	<0.001
No	99 (2.9%)	ref		

CI, confidence interval; OR, odds ratio.

a Percentages indicate the fraction of individuals who are ARPG in each specific subgroup.

## Discussion

In this study, we analyzed the prevalence of knowing about a colleague who gambles during work, being an ARPG, awareness of workplace policies, being worried about a colleague, and knowing whom to contact when worrying about a colleague. Additionally, we explored factors associated with knowing about a colleague who gambles during work and being an ARPG. The findings showed that, in this sample of both managers and subordinates from different organizations of the private and public sectors of the Swedish labor market, 4.7% of the respondents knew someone who gambles during work. However, this number should not be confused with the actual proportion of individuals who gamble during work, as some individuals may know the same gambler, which obviously decreases the frequency. Also, some gamblers may be secretive about them gambling during work, which increases the actual frequency. Previous research has found that, in a sample of transport workers, 7% gambled during work^17)^. Transport workers, along with workers in building, construction, and workers with monotonous work, generally gamble more than other occupations^1)^ This is in line with the results of this study, where ARPGs were most common in organizations mainly consisting of blue-collar workers. Of those who knew about someone gambling during work, 29 (17.4%) stated that they were worried about the negative consequences of gambling, suggesting that most employees do not worry about colleagues who gamble during work. This finding raises an important follow-up question regarding whether employees perceive gambling during work as harmful or not. Also, this suggests that employees have important information regarding gambling that is harmful to the individual and the organization, which underscore the notion that the workplace is an important area for prevention of gambling. Another noteworthy finding is that 15.2% of participants knew someone who was gaming during work, which also merits further investigation. Knowing about someone who gambles during work was significantly more common among ARPGs, men, individuals aged 25–34 years, and those knowing about a workplace gambling policy. The effect of gender is in line with previous findings, and particularly follows findings showing that workplace betting is more accepted among men^18)^. Although speculative, one reason for the relationship between knowing about a gambling policy and knowing about someone who gambles during work may related to individuals seeking out policy information when noticing that a colleague gambles during work.

The constituents of gambling or when one can be considered to be “at work” are not always evident. Uncertainties relating to when and what forms of gambling are allowed have been described in detail in interviews with Swedish sport athletes and coaches^19)^. Specifically, it was unclear to the interviewees whether the time spent traveling to and from work should be considered work or leisure time. Similar questions may arise in most workplaces. For example, is buying a scratch ticket during lunch considered to count as gambling during work? Is it appropriate for someone to stay seated at their desk during breaks and gamble on their phone? These questions underscore that gambling policies must be specific, fit the workplace context and practices, and take workplace productivity into consideration. Some might consider banning gambling during working hours as an alternative to any detailed policy. However, despite the association between workplace gambling and possible negative consequences for both employees and employers, conversations about betting on sports and placing bets during working hours can be perceived by some as peer bonding activities^20)^. Thus, implementing strict gambling policies in the workplace may cause problems if employees perceive that these policies restrict their freedom or consider them unjustified forms of punishment. If participation in sports betting pools, which is common in Sweden, during lunch breaks shifts from being overlooked to being explicitly prohibited, the result may be disgruntled employees and noncompliance with a gambling policy. Thus, employers would likely benefit from careful considerations when developing workplace policies on gambling.

ARPG constituted 3.6% of the sample. The analysis of factors related to being an ARPG showed that it was most common among those who know someone who gambles during work, men, and individuals aged 16–34 and 35–44 years. However, as two out of every five ARPGs were women, the gender differences found in this study should be used for descriptive rather than prescriptive purposes. Otherwise, there is a risk that women who are ARPGs will be overlooked. We found no difference in ARPG proportions and whether one was a subordinate or manager, or whether one knew about a workplace gambling policy.

As problem gambling is serious but of relatively low frequency, it is key to detect as many cases as possible. There are, however, multiple obstacles to detection. For example, in one study, interviewees expressed hesitation to seek help out of fear of being fired^19)^. This is likely a common fear among employees, especially those with short or insecure contracts. Thus, early detection and preventive interventions may fail simply because employees fear the consequences. This should be addressed in any workplace gambling policy by, for example, stating clearly what the employee can expect in terms of support and employment security.

This study comes with some limitations. Despite this study including a large sample of employees from different sectors and branches of the Swedish labor market, there are some limitations. The main limitation is that the sample is not nationally representative, meaning that the results do not necessarily generalize to all Swedish workplaces. Furthermore, as the organizations were recruited at a conference about harmful use, there may be selection bias. This means that the organizations interested in participating may have had more problems with harmful use than other organizations. Then again, the fact that organizational representatives participated in a conference regarding harmful use can reflect that these organizations were responsible and well-functioning than others. This may, in turn, result in an ARPG estimate that is lower than the national mean, and a knowledge of workplace gambling policy that is higher than average. Thus, selection bias may affect the estimates in different and unknown directions. However, the study included different types of workplaces and a large sample of both blue- and white-collar workers, including managers, which adds diversity. Since the present analyses are based on data collected for another purpose, the findings are exploratory. They should be further evaluated using more detailed demographics at the level of the individual. Also, respondents’ job descriptions should be included. Another limitation pertains to the response rate (42%), which may introduce bias in the estimates if there are systematic differences in response patterns related to the outcome variables. For example, if individuals who know someone who gamble during work are less likely to respond, this would result in an estimated frequency lower than the true frequency. Although the response rate is lower, it falls within the typical range of response rate in organizational research when participation is voluntary (mean 52.7%; standard deviation, 20.4%)^21)^. Moreover, we do not know the extent to which the investigated variables are associated with actual harm to the individual or the workplace. While the question about colleagues who gamble at work may act as a proxy for gambling visibility in the workplace, future research should also consider whether the respondent gambles during working hours. In larger organizations, this information can be used to prioritize the workplaces that are most in need of gambling prevention programs. For more accurate findings, future investigations of the characteristics of workplace gambling should include measures regarding the type of work done by participants, the specific gambling types utilized, whether gambling occurs during breaks or when employees should be working, the harms and benefits of gambling at work, and whether funds have been embezzled for the purpose of gambling.

## Conclusion

In conclusion, we found that 4.7% of employees in a sample of working individuals knew someone who gambled during work, and 3.5% were identified as ARPGs. Being a man, of young age, knowing about a gambling policy, and being an ARPG was associated with knowing about someone who gambles during work and being an ARPG. Similarly, ARPG was associated with being a man, aged 16–34 and 45–44 years, knowing about a gambling policy, and being a subordinate. Awareness of a workplace gambling policy was low among both subordinates and managers. Before formulating a gambling policy, organizations would benefit from exploring the gambling culture and its associated harms. Due to the heterogeneity of workplace gambling behaviors and cultures, tailoring policies and interventions to a specific organization is crucial. Future research should continue to explore any harms associated with workplace gambling and the potential positive effects perceived by employees and identify the types of workplaces that would benefit the most from gambling intervention programs.

## Data Availability

Data supporting the analysis is available on request due to privacy/ethical restrictions.
